# Antibody Response in Healthcare Workers before and after the Third Dose of Anti-SARS-CoV-2 Vaccine: A Pilot Study

**DOI:** 10.3390/vaccines10060862

**Published:** 2022-05-27

**Authors:** Alessandra Panico, Giambattista Lobreglio, Francesco Bagordo, Antonella Zizza, Antonella De Donno, Chiara Rosato, Roberta Lazzari, Michele Chicone, Floriano Indino, Virginia Recchia, Pietro Alifano, Tiziana Grassi

**Affiliations:** 1 Department of Biological and Environmental Science and Technology, University of Salento, 73100 Lecce, Italy; alessandra.panico@unisalento.it (A.P.); antonella.dedonno@unisalento.it (A.D.D.); pietro.alifano@unisalento.it (P.A.); tiziana.grassi@unisalento.it (T.G.); 2 Clinical Pathology and Microbiology Unit, Vito Fazzi General Hospital, 73100 Lecce, Italy; patologiaclinica.polecce@ausl.le.it (G.L.); chiararosato@hotmail.it (C.R.); robertaa.lazzari@alice.it (R.L.); michelechicone@hotmail.it (M.C.); floriano.indino@gmail.com (F.I.); 3 Department of Pharmacy-Pharmaceutical Sciences, University of Bari Aldo Moro, 70121 Bari, Italy; francesco.bagordo@uniba.it; 4 Institute of Clinical Physiology, National Research Council, 73100 Lecce, Italy; recchia@ifc.cnr.it

**Keywords:** SARS-CoV-2, BNT162b2 mRNA vaccine, healthcare workers, booster dose

## Abstract

The SARS-CoV-2 pandemic led to the development of various vaccines. The BNT162b2 mRNA vaccine was the first approved due to its efficacy in eliciting a humoral immunity response after the second dose. However, a decrease in the antibody concentration was observed over time. Therefore, the administration of a third dose was scheduled, primarily for frail people and workers of essential public activities. The aim of this study was to assess the level of antibodies against the spike (S) RBD of SARS-CoV-2 in healthcare workers before and after the third dose of BNT162b2 vaccine, according to sex, age, and the time interval between vaccine doses and tests. All 37 (12 males, 25 females, 19 < 50 years old, 18 ≥ 50 years old) healthcare workers recruited showed a consistent antibody titer increase after the third dose. Data analysis showed that the antibody concentration before the third dose significantly decreased as the time interval up to the test increased, and a significantly higher level was shown in young than older people. Cluster analysis revealed that young females had a higher antibody level than older females before the third dose (*p* < 0.05). This study indicated the benefit of the third dose of BNT162b2 vaccine and its effect on leveling up the humoral immune response.

## 1. Introduction

The efficacy of vaccines against Severe Acute Respiratory Syndrome Coronavirus 2 (SARS-CoV-2) is of great importance to mitigate the Coronavirus Disease 2019 (COVID-19) pandemic. The BNT162b2 mRNA vaccine (Comirnaty) was the first approved by both the Food and Drug Administration (FDA) and the European Medicines Agency (EMA), due to its efficacy in eliciting a humoral immunity response after the second dose [1,2,3]. BNT162b2, developed by Pfizer/BioNTech, is a lipid nanoparticle formulated nucleoside-modified messenger RNA (mRNA), encoding the SARS-CoV-2 spike (S) protein, stabilized in the prefusion conformation [4]. Results from clinical trials showed that this mRNA vaccine had a favorable safety profile and demonstrated 95% efficacy in preventing symptomatic COVID-19 [1,2,3].

Immunity to SARS-CoV-2 results in a decreased risk of reinfection due to the production of neutralizing antibodies [5]. Effectiveness and immunogenicity data describing the antibody kinetics overtime after natural infection, as well as vaccination, are beginning to appear [6,7], although the duration of immunity is still evolving. Breakthrough infection in people vaccinated with BNT162b2 is related to low neutralizing antibody titers [8]. However, a threshold titer capable of predicting breakthrough infection has yet to be defined. Moreover, with the diffusion of the virus variants and the relative possibility of immune escape, vaccination, in the case of infection, does not eliminate the risk of contagion but can significantly reduce the clinical consequences [9].

In the general population, the two-dose protocol for SARS-CoV-2 mRNA vaccines has been shown to elicit an excellent antibody response [10]. However, several studies compared the IgG levels at different timepoints after vaccination and highlighted a decrease in antibody concentration a few months after the second dose of vaccine, indicating a waning of the immune response over time [11,12,13,14]. Therefore, there is great concern regarding the weakened SARS-CoV-2 immune protection.

In order to counter this decline in antibody concentration and keep the immune defenses high in the population, the FDA, on 22 September 2021, authorized an additional dose of BNT162b2 vaccine ≥ 6 months after completion of the primary series among persons aged ≥ 65 years or whose occupational or institutional exposure puts them at high risk for COVID-19 [15]. A few days later, the EMA authorized booster doses at least 6 months after the second dose for people aged 18 years and older [16].

Therefore, many countries are proceeding with the administration of the third dose of vaccine (booster dose), starting with frail people, who are at greater risk of developing severe symptoms in case of contact with the virus, and people who carry out essential public activities such as healthcare workers, public security forces, and teachers.

In the present pilot study, the level of antibodies against the receptor-binding domain of the SARS-CoV-2 spike protein (against the spike (S) RBD) of SARS-CoV-2 in healthcare workers was assessed before and after the third dose of BNT162b2 vaccine. The antibody level was analyzed according to sex, age, and the time interval between vaccine doses and testing.

## 2. Materials and Methods

### 2.1. Study Design and Participants

This pilot study was part of the “COVID-19 Research Project” promoted by the Local Health Authority (ASL) of Lecce and the University of Salento. In the period between 25 September 2021 and 7 January 2022, a group of twice vaccinated healthcare workers were screened for antibodies against the spike (S) RBD of SARS-CoV-2 at the Clinical Pathology and Microbiology Unit of the “Vito Fazzi” Hospital in Lecce (Puglia, Italy). The screening was performed before and after the third dose of the BNT162b2 vaccine. At the same time, the cohort was also screened for antibodies against the nucleoprotein (N) of SARS-CoV-2, in order to reveal a previous SARS-CoV-2 infection. None of these participants worked in the COVID-19 unit.

Inclusion criteria for participation in this study were the following: (1) twice vaccinated; (2) free from chronic diseases (cancer, autoimmune disorders, etc.); (3) absence of previous SARS-CoV-2 infection (clinically, radiologically, or by laboratory results); (4) signed informed consent form.

### 2.2. Data Collection

For each subject, the age, sex, vaccination status (number of doses, dates, and type of vaccine), and dates of blood draws for serological tests were recorded. A signed informed consent form was obtained from all participants for research data collection.

### 2.3. Detection of Antibodies against Spike (S) RBD

The determination of antibodies against the spike (S) RBD of SARS-CoV-2 was carried out in serum samples using the Elecsys^®^ Anti-SARS-CoV-2 S (Roche Diagnostics GmbH, Mannheim, Germany), an electrochemiluminescence immunoassay (ECLIA) developed for the in vitro quantitative detection of total antibodies (including immunoglobulin G) against the SARS-CoV-2 S protein RBD in human serum and plasma. The assay uses a recombinant protein representing the RBD of the S protein in a double-antigen sandwich assay format, which favors the detection of high-affinity antibodies against SARS-CoV-2. According to the manufacturer’s instructions, the measuring range spanned from 0.4 to 250 U/mL (up to 25,000 U/mL with onboard 1:100 dilution on a Cobas 8000 analyzer); values higher than 0.8 U/mL were considered positive.

### 2.4. Detection of Antibodies against Nucleoprotein (N)

Antibodies targeting both the spike (S) and the nucleocapsid (N) proteins are detected in individuals previously infected by SARS-CoV-2. In this study, the Elecsys^®^ Anti-SARS-CoV-2 N (Roche Diagnostics GmbH, Mannheim, Germany), which uses a recombinant protein representing the N antigen in a double-antigen sandwich assay format, was used to identify subjects with a history of previous SARS-CoV-2 infection and to exclude immunization due to infection rather than vaccination.

### 2.5. Statistical Analysis

Data on subjects’ details and serological analysis were entered in a Microsoft Excel database and statistically analyzed using MedCalc Software version 12.3 (MedCalc Software bvba, Ostend, Belgium).

Descriptive statistics for baseline population characteristics were calculated as means, standard deviations, and frequencies (%). In addition, to graphically compare the results of the different groups, the data were represented through a boxplot showing the distribution of antibody concentration as the interquartile range (each segment and rectangle) along with the minimum, first quartile, median, third quartile, and maximum, as well as any outliers.

Any differences in humoral responses between groups were assessed by the nonparametric Kruskal–Wallis test, since a normal distribution of values could not be assumed due to the small sample size. In addition, a clustered multiple comparison analysis for gender and age group was performed to identify any cluster within the screened population with significant differences in antibody titer.

Lastly, simple linear regression was performed to verify whether the antibody level was dependent on the time (days) since the last vaccine dose.

### 2.6. Ethical Aspects

The study was approved by the Ethical Committee of the Lecce Local Health Authority (ASL/LE) on 29 May 2020 with deliberation n. 557. All data were collected and analyzed confidentially in accordance with Italian laws (Legislative Decree n. 196 of 30 June 2003, and subsequent additions) for research purposes.

## 3. Results

Table 1 reports the study population characteristics. Thirty-seven healthcare workers were recruited in the study. Among them, 25 (67.6%) were females and 12 were males. The average age was 45.5 ± 12.5 years, and 18 subjects (48.6%) were ≥50 years old. None of them had previously contracted COVID-19, resulting negative for antibodies against N, and all tested positive for antibodies against the spike (S) RBD before and after the third dose of vaccine.

**Figure 1:**
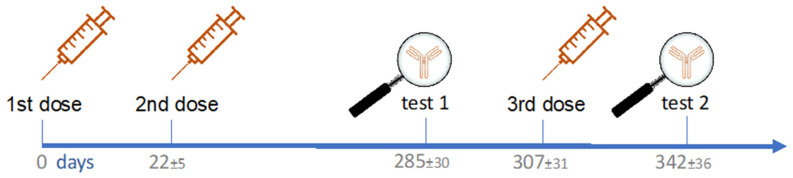
Timeline of vaccine doses and testing with mean time interval ± SD (days).

The mean time interval between the first and the second dose was 22 ± 5 days, that between the first and the third dose was 307 ± 31 days, that between the second dose and Test1 was 262 ± 33 days, and that between the third dose and Test2 was 35 ± 17 days. Figure 1 shows the timeline of vaccine doses and testing since the first dose.

**Figure 2:**
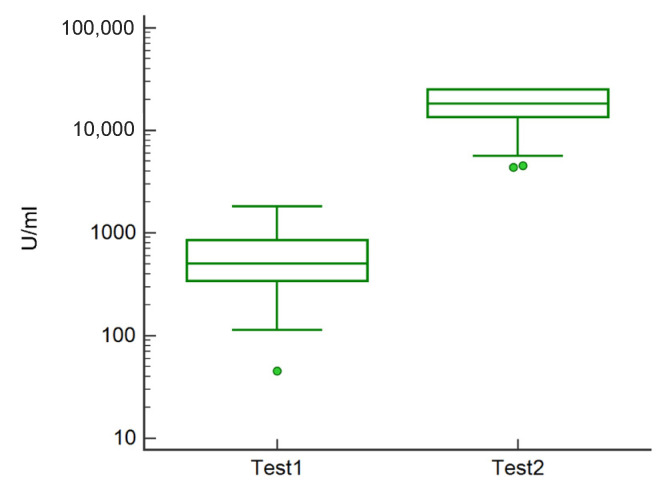
Boxplot showing the distribution of antibody concentration in analyzed subjects before (Test1) and after the third dose (Test2). Green circles represent the outliers.

In general, the mean antibody titer was 652.2 ± 450.7 U/mL before the third dose and 17,446.8 ± 7323.7 U/mL after the third dose, with an average increase in antibodies of about 27-fold after the third dose (Test2) (Figure 2).

Before the third dose, people aged <50 years showed a mean antibody titer significantly higher (*p* < 0.05) (801.1 ± 430.2 U/mL) than subjects ≥ 50 years (495.0 ± 450.7 U/mL). Female participants highlighted an antibody concentration of 697.0 ± 447.6 U/mL, while males highlighted an antibody concentration of 558.7 ± 431.0 U/mL.

**Figure 3:**
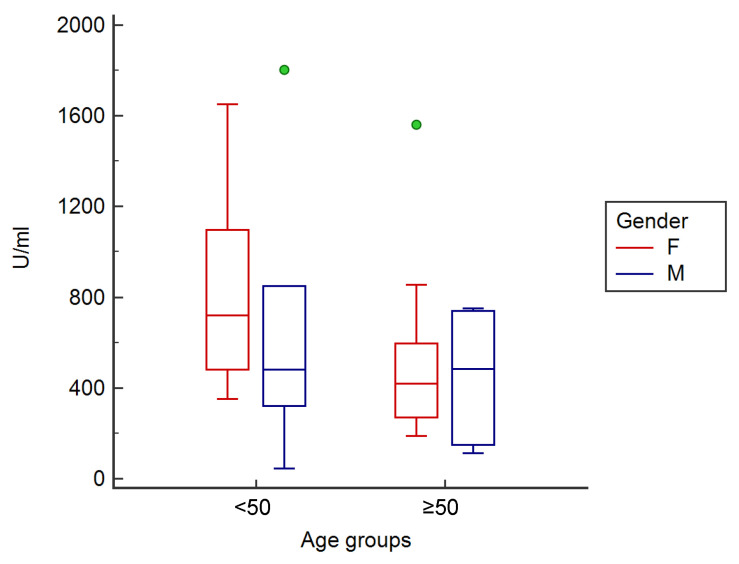
Clustered multiple comparison graph showing the minimum, maximum, median, and outliers (green circles), as well as the distribution of antibody concentration as an interquartile range (each segment or rectangle) in analyzed subjects according to gender and age group before the third dose of vaccine.

Cluster analysis for gender and age group showed that, before the third dose of the vaccine, young (<50 years old) female subjects (*n* = 13, 864.5 ± 386.5 U/mL) had a higher antibody level than old female subjects (*n* = 12, 515.7 ± 447.6 U/mL) (*p* < 0.05), as well as male subjects, both <50 (*n* = 6, 663.7 ± 443.8 U/mL) and ≥50 years (*n* = 6, 453.7 ± 354.1 U/mL) (Figure 3).

After the third dose, people < 50 years showed a mean antibody titer of 19,459.3 ± 7341.4 U/mL, subjects ≥ 50 years showed a mean antibody titer of 15,322.6 ± 7323.7 U/mL, female participants showed a mean antibody titer of 18,166.4 ± 7384.7 U/mL, and males showed a mean antibody titer of 15,947.8 ± 7205.9 U/mL.

**Figure 4:**
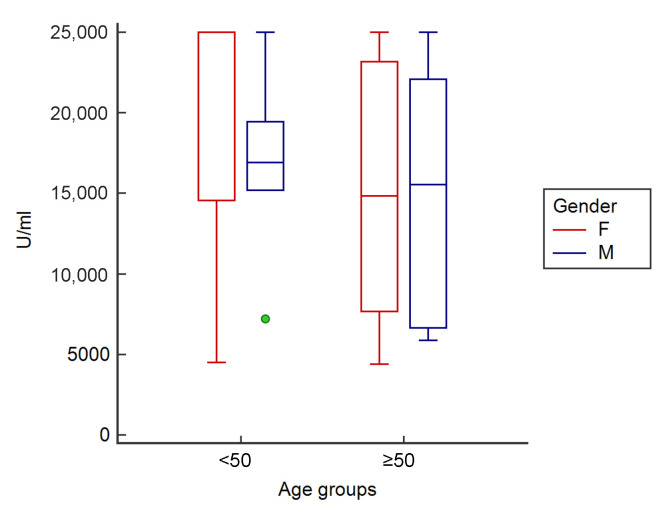
Clustered multiple comparison graph showing the minimum, maximum, median, and outliers (green circles), as well as the distribution of antibody concentration as an interquartile range (each segment or rectangle) in analyzed subjects according to gender and age group after the third dose of vaccine.

Cluster analysis for gender and age group showed that, after the third dose, young (<50 years old) female subjects (*n* = 13, 20,698.2 ± 7447.0 U/mL) also had a higher antibody level than male subjects, both <50 (*n* = 6, 16,775.0 ± 7269.2 U/mL) and ≥50 years (*n* = 6, 15,120.5 ± 7003.7 U/mL), as well as old female subjects (*n* = 12, 15,423.6 ± 7384.7 U/mL) (Figure 4).

**Figure 5:**
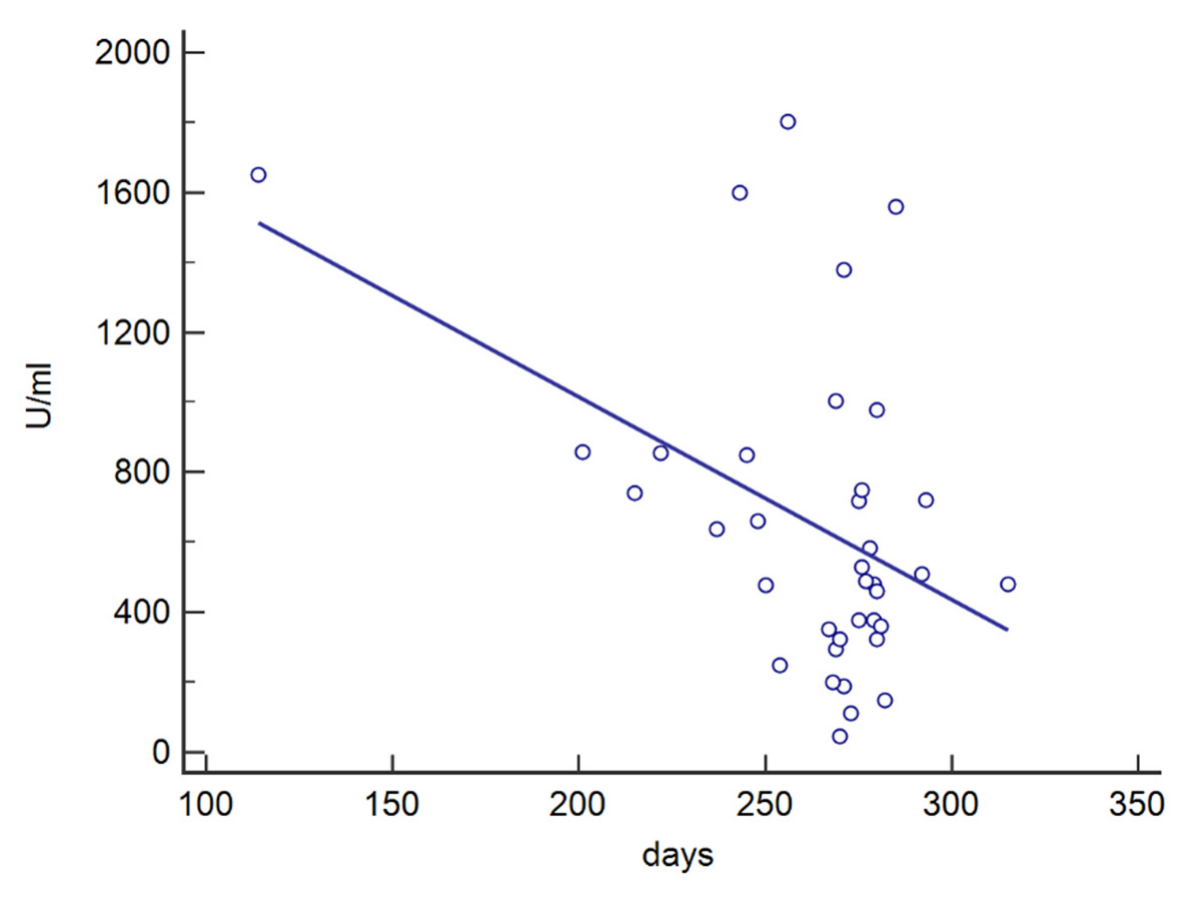
Linear correlation between the level of antibodies detected at Test1 and the time (days) elapsing from the second dose.

The influence of the time interval between vaccine doses and testing for antibody titers was also investigated. In particular, the antibody concentration detected at Test2 resulted independent from the time interval between the second and the third dose (*p* = 0.904, *r*^2^ < 0.001) and the time interval between the third dose and Test2 (*p* = 0.195, *r*^2^ = 0.048). In contrast, the antibody titer observed at Test1 resulted dependent from the time interval between the second dose and Test1 (*p* = 0.008, *r*^2^ = 0.186), with a decrease in the antibody concentration as the time interval increased (Figure 5).

## 4. Discussion

The repeated waves of the COVID-19 pandemic have highlighted the necessity to optimize vaccine responses in the population, especially in vulnerable groups and in people exposed to the risk of contracting the disease because of their occupation, such as healthcare workers.

In this study, the level of antibody against the spike (S) RBD of SARS-CoV-2 was assessed in healthcare workers before and after the third dose of BNT162b2 vaccine.

After the second dose, the humoral immune response seemed to be age-dependent, with a more evident reaction in young women.

We observed that the BNT162b2 mRNA vaccine is particularly effective in producing a high antibody titer against the SARS-CoV-2 spike (S) RBD after the booster dose (27-fold higher). This antibody increase produced a leveling of the antibody titer and, although the young women always showed a slightly higher concentration than the other clusters, no significant differences by gender and age were observed after the third dose.

Several studies investigated the relationship among age, gender, and level of antibody against SARS-CoV-2 after BNT162b2 vaccination. Levin et al. [12] observed that, 6 months after receipt of the second dose, the humoral response was substantially decreased, especially among men, persons 65 years of age or older, and patients with immunosuppression. The same evidence was also found following the administration of a single dose of vaccine in a large cohort (*n* = 8837) for both BNT162b2 and ChAdOx1 vaccines [17]. Another study found that the age of vaccinated individuals was significantly negatively correlated with S-RBD IgG response, with a lower antibody concentration as the age increased [13]. Pellini et al. [18] observed that, after two BNT162b2 vaccine doses, the antibody response was of greater magnitude in women and young participants vs. men and old participants. Our study also highlighted this difference following the third dose of BNT162b2 vaccine, despite the small cohort.

Vaccine responses may be influenced by age, with older people developing lower antibody levels and cell-mediated responses. Furthermore, such individuals generally had a more rapid antibody decline after vaccination [19].

A recent meta-analysis highlighted differences in morbidity and mortality between sexes in infected COVID-19 subjects. Male patients had higher odds of admission to the intensive treatment unit and of death than females [20]. This difference was confirmed in our results of vaccine response.

Clinical data illustrated that males and females differ in vaccine-induced immune responses, adverse events, and protection [21]. Women typically develop higher antibody responses after vaccination against influenza, rubella, measles, mumps, hepatitis A and B, etc. [22].

Therefore, women seem to react more strongly to immunization, but also report more side-effects than males. According to a report by the Centers for Disease Control and Prevention on COVID-19 vaccine safety monitoring [23], 79.1% of vaccine-related adverse reactions were reported by women. Naaber et al. [13] observed that the presence and the level of side-effects appear to be related to the strength of the humoral response.

Sex-based differences in humoral immune responses may be due to genetic and hormonal factors. Females generally show a greater antibody response, a larger number of B cells, and a higher level of basal immunoglobulin [24,25,26]. Global gene expression analysis of B cells detected a significant upregulation of most differentially expressed genes between sexes in females compared with males [27]. Moreover, many genes of the X chromosome are involved in immune function regulation, which may play an important role in sex-specific responses to vaccines [28]. Lastly, sex hormones, such as testosterone, estradiol, and progesterone, may also influence the immune response to vaccines. In particular, an immunosuppressive role of testosterone was proven in the response to influenza [24] and to COVID-19 vaccination [29].

Our study showed that older female participants exhibited a lower antibody concentration than younger females both before and after the third dose of vaccine. This could be explained by the established evidence that antibody responses are lower in aged individuals than in their younger counterparts [30,31], whereas sex differences in the immune response may depend, as well as on the aforementioned genetic and hormonal factors, on the specific vaccine, as demonstrated by other studies [21].

The linear correlation between the antibody level detected at Test1 and the time since the second dose showed a decrease in the antibody concentration as the time interval increased. The booster dose elicited a consistent increase in the antibody titer for all participants and, considering the low level of the humoral response several months after the second dose, we can also speculate a similar trend for the third dose.

A recent survey demonstrated a high correlation between IgG against the RBD and neutralizing antibody titers, suggesting that IgG might serve as a correlate of neutralization [32]. However, since correlates of protection have not yet been determined, we are unable to establish the antibody level needed for protection. Further studies are required to define correlates of protection and evaluate the clinical significance of this occurrence. Moreover, it is important to consider that other immune responses, such as T-cell-mediated immunity, contribute to vaccination efficacy [33].

This study is not without limitations, such as the small number of participants that allowed obtaining only preliminary results, and the lack of accurate serological monitoring over time from the first dose of vaccine which would have allowed us to investigate the antibody kinetics. Moreover, the male population was under-represented, while females were over-represented; hence, conclusions about gender differences in vaccine response need further confirmation. Nevertheless, our cohort represents healthcare workers, a group highly exposed to COVID-19, who are typically younger and healthy populations, with a higher proportion of females.

On the other hand, despite the restricted number of participants, young female subjects showed a higher antibody concentration than the other clusters, at both Test1 and Test2, underling the reliability of this result. Moreover, the findings that emerged in this study can be useful to define less reactive populations that might be more susceptible to breakthrough infections, and they underline the need to constantly monitor the vaccination response. In fact, the cohort analyzed in the present study continues to be monitored and expanded in order to measure the antibody response over time and identify additional factors that can influence it. Hence, our ongoing study has the potential to provide critical information on the duration of the BNT162b2 mRNA vaccine-mediated protection.

## 5. Conclusions

In this study we found that all recruited healthcare workers, without a history of COVID-19, developed antibodies against the spike (S) RBD following three doses of the BNT162b2 vaccine. The analysis of humoral immune response allowed highlighting the following main findings:

- the antibody concentration after the second dose decreased as the time interval up to test increased;
- the antibody concentration after the second dose was age-dependent with a significantly higher level in young people than in older ones;
- the third dose of vaccine induced a robust antibody response in all participants with respect to the previous antibody levels;
- young female subjects showed a higher antibody concentration both before and after the third dose.

The assessment of humoral response against SARS-CoV-2 over time is fundamental to estimate the duration of protection and to evaluate the variables that are useful to determine the need for further boosters to protect people.

## Figures and Tables

**Table 1:** Characteristics of the study cohort, antibody titers, and time intervals between vaccine doses and testing.

Variable		
Gender		
Male	12 (32.4)	N (%)
Female	25 (67.6)	N (%)
Age	45.5 ± 12.5	Mean ± SD (years)
<50	19 (51.4)	N (%)
≥50	18 (48.6)	N (%)
Antibody titer after the 2nd dose (Test1)	652.2 ± 450.7	Mean ± SD (U/mL)
Antibody titer after the 3rd dose (Test2)	17,446.8 ± 7323.7	Mean ± SD (U/mL)
Interval 1st–2nd dose	22 ± 5	Mean ± SD (days)
Interval 1st–3rd dose	307 ± 31	Mean ± SD (days)
Interval 2nd–3rd dose	284 ± 35	Mean ± SD (days)
Interval 2nd dose–Test1	262 ± 33	Mean ± SD (days)
Interval Test1–3rd dose	22 ± 17	Mean ± SD (days)
Interval 3rd dose–Test2	35 ± 17	Mean ± SD (days)

## Data Availability

The data presented in this study are available on request from the corresponding author.
